# Cumulative Lifetime Violence and Bacterial Vaginosis Infection in Sexually Transmitted Infections: Findings From a Retrospective Cohort Study Among Black Women at Risk for HIV

**DOI:** 10.1016/j.focus.2023.100180

**Published:** 2023-12-24

**Authors:** Yordanos Tesfai, Marguerite B. Lucea, Erica Chan, Theresa Asuquo, Helen Zhu, Tommi L. Gaines, Jacquelyn C. Campbell, Jamila K. Stockman, Kiyomi Tsuyuki

**Affiliations:** 1 Family Medicine and Public Health, University of California, San Diego, La Jolla, California; 2 Department of Nursing, College of Health Professions, Towson University, Towson, Maryland; 3 Program in Medical Education - Health Equity (PRIME-HEQ), Department of Medicine, University of California, San Diego, La Jolla, California; 4 Division of Infectious Diseases & Global Public Health, Department of Medicine, School of Medicine, University of California, San Diego, La Jolla, California; 5 School of Nursing, Johns Hopkins University, Baltimore, Maryland

**Keywords:** Bacterial vaginosis, sexually transmitted infections, cumulative violence, physical abuse, sexual abuse, Black women

## Abstract

**Introduction:**

Bacterial vaginosis is the most common vaginal condition among women of reproductive age and has been associated with sexually transmitted infections. This study examines the association between cumulative lifetime violence exposure, bacterial vaginosis, and sexually transmitted infections among Black women at risk for HIV.

**Methods:**

HIV-negative Black women in a retrospective cohort study (N=230) completed survey questions on cumulative violence (exposure to sexual or physical abuse before age 18 years and exposure to intimate partner violence or sexual violence [partner or other] after age 18 years and past year), bacterial vaginosis (lifetime and past year), and sexually transmitted infection diagnosis (lifetime and past year). Logistic regression models estimated the associations between cumulative violence, bacterial vaginosis, and sexually transmitted infections. Bacterial vaginosis was examined as a moderator in the association between cumulative violence and sexually transmitted infections.

**Results:**

Many women reported cumulative violence exposure (40%), lifetime bacterial vaginosis diagnosis (53%), and lifetime sexually transmitted infection diagnosis (73%). Cumulative violence experience was significantly associated with increased adjusted odds of lifetime bacterial vaginosis diagnosis (AOR=1.98; 95% CI=1.10, 3.54). Lifetime bacterial vaginosis diagnosis (AOR=2.76; 95% CI=1.45, 5.22) and past-year bacterial vaginosis diagnosis (AOR=2.16; 95% CI=1.14, 4.10) were significantly associated with increased odds of lifetime sexually transmitted infection diagnosis. Lifetime bacterial vaginosis diagnosis (AOR=2.10; 95% CI=1.19, 3.70) and past-year bacterial vaginosis diagnosis (AOR=3.00; 95% CI=1.70, 5.31) were significantly associated with past-year sexually transmitted infection diagnosis. Lifetime bacterial vaginosis infection significantly increased the odds of lifetime sexually transmitted infection diagnosis with increasing cumulative violence exposure.

**Conclusions:**

Our findings support educating and screening Black women who experience cumulative violence for bacterial vaginosis to reduce the risk of untreated bacterial vaginosis and sexually transmitted infections.

## HIGHLIGHTS

• Bacterial vaginosis (BV) and sexually transmitted infections (STI) are disparately prevalent among Black women.
• Cumulative violence experience was significantly associated with increased adjusted odds of lifetime BV diagnosis.
• Lifetime BV diagnosis and past-year BV diagnosis were significantly associated with increased odds of lifetime STI diagnosis. Lifetime BV diagnosis and past-year BV diagnosis were significantly associated with past-year STI diagnosis.
• More education and support are needed for Black women who experience cumulative violence for BV to reduce the risk of untreated BV and STIs.

## INTRODUCTION

In the U.S., Black women are disproportionately affected by sexually transmitted infections (STIs), including HIV.^1^ In 2019, the rate of reported cases of chlamydia, gonorrhea, and syphilis among Black women was 5.2, 6.8, and 4.4 times, respectively, the rate among White women.^2^ In 2019, Black women had the highest HIV prevalence (57%) and the highest rate of new HIV diagnoses (22.2 per 100,000) among women.^3^^,^^4^ Ulcerative and inflammatory STIs significantly increase the efficiency of HIV transmission by increasing both one's infectiousness of HIV and one's susceptibility to HIV infection if exposed.^5^

Bacterial vaginosis (BV) is a lesser understood inflammatory infection of the female reproductive tract. It is the most common vaginitis among women of reproductive age (15–44 years), affecting an estimated 29% of women aged 14–49 years in the U.S.^6^ Many women are asymptomatic for BV, but a frequent symptom is thin, odorous discharge. Although the causes of BV are not fully understood, factors associated with an increase in BV infection include being pregnant, practicing vaginal douching, having sex with women, engaging in risky sexual behaviors (e.g., condomless sex), and reporting high stress levels.^7^, ^8^, ^9^

BV is associated with an increased risk of female sexual and reproductive health conditions, such as preterm birth, STIs (including HIV), and increased HIV viral shedding.^5^^,^^10^, ^11^, ^12^, ^13^, ^14^, ^15^ The mechanisms linking BV and HIV/STI incidence are the subject of ongoing research. Some research posits that herpes simplex virus Type 2 is a risk factor for BV and HIV acquisition.^16^ Other research highlights that through an imbalance of the vaginal flora, BV heightens the inflammatory state of the vaginal epithelium, resulting in the recruitment of immune cells vulnerable to HIV infection in the cervix.^5^^,^^17^

Black women experience a disproportionate prevalence of BV compared with their racial and ethnic counterparts.^6^^,^^17^ This disparity persists after controlling for demographic and behavioral factors (e.g., age of sexual initiation).^6^^,^^18^ National studies consistently find that Black women report twice the BV prevalence of White women.^8^ Although limited studies investigate the connection between BV and STI infection, some posit that BV may fuel STI and HIV disparities among Black women in the U.S.^17^

In addition, stress is consistently linked to increased BV risk. Moderate to high levels of psychosocial stress were associated with increased risk for BV among women, adjusting for education, number of sexual partners, age, race and ethnicity, use of illicit drugs, and vaginal douching practices.^19^^,^^20^ Data from the Longitudinal Study of Vaginal Flora indicated that for each point increase in perceived stress score, women had 29% increased odds of BV diagnosis, net of risk behaviors.^21^^,^^22^

Violence across the life course is an important chronic stressor for all survivors. As a chronic stressor, cumulative lifetime violence exposure can negatively impact health outcomes more than a single violence exposure.^23^ Black women are disproportionately affected by sexual, physical, and psychological abuse compared with White women.^22^ However, little research currently frames cumulative lifetime violence as a chronic stressor,^24^ and limited research assesses the association between cumulative lifetime violence and BV and STIs among Black women in the U.S.

We apply the minority stress theory (MST) with the concept of toxic stress to guide our examination of the connection between violence and both BV and STIs among Black women.^25^ MST posits that individuals are embedded within sociostructural contexts, which determine the stress they encounter and their coping resources. This theory provides a practical framework to explain how health disparities based on racial, gender, and socioeconomic inequity confer stress–immune dysregulation and enhanced vulnerability to infection.^26^ According to MST, Black women confront a disproportionate amount of cumulative violence and stress from their intersecting minority stressors (e.g., gender, race) and have fewer coping resources to manage the violence-related stress than cisgender, heterosexual White men and women, which leads to adverse physical and mental effects.^25^^,^^27^

Toxic stress posits that early stress experiences are built into our bodies, creating a vulnerability to future stressors. This chronic stress produces physiologic disruptions or biological memories that undermine the body's stress response systems and affect the developing brain, immune system, and other regulatory controls.^28^, ^29^, ^30^ Together, minority stress and toxic stress posit associations between increased exposure to cumulative lifetime violence and increased susceptibility of Black women to BV infection and other STIs.^25^^,^^27^

To our knowledge, despite robust findings linking stress and BV, no study has examined violence as a potential chronic stressor and risk factor for BV and STIs among Black women. Yet, trauma and violence have long-lasting consequences on sexual and reproductive health. This study examines the associations across cumulative violence, BV infection, and STIs among a sample of Black women at high risk for HIV. We hypothesize that lifetime cumulative violence exposure is positively and significantly associated with BV and STI diagnosis. In addition, given prior work suggesting that BV increases STI risk, we also hypothesize that the presence of a BV diagnosis strengthens (moderates) the association between cumulative violence and STIs.

## METHODS

### Study Sample

The data analyzed is from the ESSENCE Project: Examining Stress, Sexual Experiences, and Neighborhood Correlates of HIV Risk among Black Women (NICHD R01HD077891). The ESSENCE Project is a retrospective cohort study (2015–2018) examining the associations between the built and social environment, sexual assault, and HIV risk factors among Black women in Baltimore, Maryland. Black women seeking services were recruited in the waiting rooms of 2 Baltimore City public sexually transmitted disease clinics. After providing informed consent, participants were screened for eligibility using the following inclusion criteria: biologically female, aged between 18 and 44 years, self-identified as Black, reported having sex with a man in the past 6 months, and reported having either 2 or more sexual partners in the past year or a high HIV risk sexual partner (i.e., used injection/noninjection drugs, had sex with men, been to prison, had concurrent sex partner, had a sexually transmitted disease, was HIV positive, or did not know whether their sexual partners had any of these characteristics). By study design, at least one third of women reported *sexual violence exposure*, defined as someone insisting on having sex; insisting on sex without a condom; or using force or threats to engage in oral, vaginal, or anal sex since age 18 years (hence referred to as exposed). Of the 940 women screened for participation in ESSENCE, 310 women were eligible and given the survey. These women proceeded to full survey completion (60–90 minutes), which was administered through audio computer-assisted self-interview. Each woman received $10 for eligibility screening, $25 for completing the survey, and a list of local community resources. The ethical review boards of Johns Hopkins University and the University of California, San Diego approved the study design and procedures. An NIH Certificate of Confidentiality was issued to protect our study's research participants' privacy.

### Measures

**Lifetime and past-year bacterial vaginosis diagnosis**. Our primary dependent variable was self-reported. Women were asked whether a doctor had told them in their lifetime or the past 12 months that they had BV.

**Lifetime and past-year sexually transmitted infection diagnosis**. Our secondary dependent variable was measured on the basis of whether a doctor told women that they had chlamydia, gonorrhea, trichomoniasis, syphilis, herpes, hepatitis B, and/or hepatitis C in their lifetime or within the past 12 months. If a woman reported having any STI(s) within the specified time frame, they were considered to have a positive STI status in their lifetime and/or within the past year.

**Cumulative violence**. Our primary independent variable was a summative measure of the lifetime experience of violence occurring before the age of 18 years, after the age of 18 years, and in the past 12 months. We measured physical and sexual violence before the age of 18 years, intimate partner violence (IPV) (physical/psychological/sexual) and nonintimate partner sexual violence after the age of 18 years, and IPV and nonintimate partner sexual violence in the past 12 months. A woman could score a maximum of 2 points for violence in each life course stage for a maximum score of 6. Violence before the age of 18 years was determined by asking *Before you turned 18 (anytime during your childhood or teen years), has anyone done the following: 1) Physically abused you (by physical abuse, we mean slapping, beating, kicking, choking, or threats with weapons)? 2) Sexually abused you (by sexual abuse, we mean forcing or pressuring for sex or physically hurting the sexual parts of your body, including touching that made you uncomfortable)?* Violence after the age of 18 years was determined through several questions. IPV included responses to the questions *Since you turned 18, has a male sex partner: 1) Been very jealous and controlling? Done something to make you afraid of him? Punched, kicked, slammed you against the wall, or beat you up? Choked you? and/or 2) Used threats to make you have sex when you did not want to? Used force (like hitting, holding down, or using a weapon) to make you have sex?* Nonintimate partner sexual violence was determined by asking *Since you turned 18, has any other male: 1) used threats to make you have sex when you did not want to?* and/or 2) *used force (like hitting, holding down, or using a weapon to make you have sex?* Violence in the prior 12 months was determined using specific Conflict Tactics Scales items.^31^ *Physical violence by an intimate partner or other person* was defined as threatening to hit or throw something, twisting arm or hair, pushing or shoving, grabbing, slapping, using a weapon, punching, choking, beating, burning, and kicking. To determine IPV in the last 12 months, we also asked whether a partner had *been very jealous or controlling* or *done something to make you afraid of them* in the past year. *Sexual violence by an intimate partner or other person* was defined as insisting on having sex; insisting on sex without a condom; or using force or threats to engage in oral, vaginal, or anal sex. A binary cumulative violence variable was created to assess the presence of at least 1 form of cumulative violence.

### Statistical Analysis

All statistical analyses were conducted using Stata, Version 15.^32^ Listwise deletion was performed on the 310 women to include individuals with complete data, resulting in a final analytic sample of 230 women. Upon further investigation, women included in the analysis were similar to women excluded from the analysis on most sociodemographics except for more women who reported current unstable housing were excluded from the analysis.

We began our analysis with univariate descriptive statistics of sociodemographic characteristics, the independent variable of cumulative violence, and the dependent variables of BV diagnosis and STI diagnosis (Table 1). We then compared sociodemographic characteristics, cumulative violence, and STI diagnosis by lifetime and past-year BV. We then estimated bivariate logistic regression models. First, we separately estimated the associations between cumulative violence (independent variable and each type of abuse included in cumulative violence measure) and lifetime BV diagnosis (Table 2: Models 1–8) and past-year BV diagnosis (Table 2: Models 9–16); testing the hypothesis that cumulative violence is associated with greater odds of BV infection. Second, we separately estimated the associations between cumulative violence and BV infection on lifetime STI diagnosis (Table 3: Models 1–4) and past-year STI diagnosis (Table 3: Models 6–9), testing the hypothesis that cumulative violence and BV infection is associated with greater odds of STI. Bivariate logistic regression models with significant associations were adjusted for age, education, and total number of lifetime sexual partners. We applied the false discovery rate (FDR) using the Benjamini and Hochberg method to account for multiple comparisons and report original and adjusted *p*-value estimates using a 0.05 FDR level for models in Tables 2 and 3.^33^

**Table 1:** Sociodemographic Characteristics, Violence, and STIs by BV Diagnosis Among Black Women in Baltimore, Maryland (N=230)

	Lifetime BV					Past-year BV					Total	
Variables	No		Yes		*p*-value^a^	No		Yes		*p*-value^a^		
	*n*=107	47%	*n*=123	53%		*n*=130	57%	*n*=100	43%		*n*=230	100%
Sociodemographics												
Age,^b^ years	24	(20–28)	27	(23–32)	**0.000**⁎⁎⁎	25	(20–29)	27	(22–32)	**0.026**⁎	25	(21–30)
High school education or more	90	84	104	85	0.927	111	85	83	83	0.622	194	84
Formally employed	70	65	71	58	0.232	82	63	59	59	0.529	141	61
Unstable housing	6	6	8	7	0.777	9	7	5	5	0.545	14	6
Number of children^b^	0	(0–2)	1	(0–2)	0.586	1	(0–2)	1	(0–2)	0.926	1	(0–2)
Individual income					0.618					0.083		
<$10,000	66	62	68	55		84	65	50	50		134	58
$10,000–$29,999	32	30	43	35		36	28	39	39		75	33
>$30,000	9	8	12	10		10	8	11	11		21	9
Relationship status					0.643					0.448		
Single	72	67	86	70		86	66	72	72		158	69
In relationship	30	28	34	28		38	29	26	26		64	28
Separated/divorced/widowed/other	5	5	3	2		6	5	2	2		8	3
Sexual orientation					0.115					0.303		
Heterosexual	95	89	100	81		113	87	82	82		195	85
Bisexual/lesbian/gay	12	11	23	19		17	13	18	18		35	15
Sexual partners (total number)^b^	2.00	(1–2)	2.00	(1–3)	0.087	2.00	(1–2)	2.00	(1–3)	0.299	2.00	(1–2)
Violence												
Cumulative violence^b^	0	(0–3)	0	(0–3)	**0.003**⁎⁎	0	(0–4)	0	(0–4)	**0.031**⁎	0.00	(0–4)
Cumulative violence (report ≥1)	31	29	60	49	**0.002**⁎⁎	43	33	48	48	**0.022**⁎	91	40
Child sexual abuse (ever)	21	20	35	28	0.120	30	23	26	26	0.609	56	24
Child physical abuse (ever)	19	18	37	30	**0.030**⁎⁎	29	22	27	27	0.411	56	24
Adult sexual abuse (ever)	31	29	60	49	**0.002**⁎⁎	43	33	48	48	**0.022**⁎	91	40
Adult physical abuse (ever)	25	23	51	41	**0.004**⁎⁎	36	28	40	40	**0.049**⁎	76	33
Adult sexual abuse (past year)	18	17	27	22	0.328	24	18	21	21	0.630	45	20
Adult physical abuse (past year)	17	16	26	21	0.308	21	16	22	22	0.260	43	19
STIs												
STIs (number ever)^b^	1	(0–1)	2	(1–3)	**0.000**⁎⁎⁎	1	(1–2)	2	(1–3)	**0.001**⁎⁎⁎	1	(1–2)
Yes	67	62	102	83	**0.001**⁎⁎⁎	87	67	82	82	**0.010**⁎⁎	169	73
STIs (number in past year)^b^	0	(0–1)	1	(0–2)	**0.038**⁎	0	(0–1)	1	(0–2)	**0.001**⁎⁎⁎	0	(0–1)
Yes	41	38	65	53	**0.027**⁎	47	36	59	59	**0.001**⁎⁎⁎	106	46

*Source:* The ESSENCE Project 2015–2018.

*Note:* Boldfaces indicate statistical significance.

⁎ *p<*0.05, **p<0.01, and ***p<0.001).

a *p*-value for statistical difference between BV infection and no BV infection using chi-square for comparisons among categorical variables (unless there is a zero cell) and the F-statistic for comparison of means among continuous variables.

b Median (IQR) reported for continuous variables; columns may add to >100% owing to rounding. BV, bacterial vaginosis; STI, sexually transmitted infection.

**Table 2:** Logistic Regression Models of Cumulative Violence and Violence Types as Predictors of BV Infection Among Black Women in Baltimore, Maryland (N=230)

Model		Lifetime BV (yes/no)								Model	Past-year BV (yes/no)							
		Unadjusted model				Adjusted model					Unadjusted model				Adjusted model			
		OR	95% CI	*p*-value	Adjusted *p*-value	AOR	95% CI	*p*-value	Adjusted *p*-value		OR	95% CI	*p*-value	Adjusted *p*-value	AOR	95% CI	*p*-value	Adjusted *p*-value
1	Cumulative violence (continuous)	1.17	(1.03, 1.33)	**0.015**⁎	**0.030**⁎	1.12	(0.98, 1.28)	0.090	0.113	9	1.10	(0.97, 1.24)	0.129	0.258	—			
2	Cumulative violence (categorical)									10								
	0	ref					ref				ref					ref		
	1–6	2.33	(1.35,4.04)	**0.002**⁎⁎	**0.005**⁎⁎	1.98	(1.10, 3.54)	**0.021**⁎	0.053		1.87	(1.09, 3.19)	**0.022**⁎	0.088	1.61	(0.92, 2.81)	0.095	0.143
3	Child sexual abuse (ever)	1.63	(0.88, 3.02)	0.121	0.161	—				11	1.17	(0.64, 2.14)	0.609	0.631	—			
4	Child physical abuse (ever)	2.00	(1.06, 3.73)	**0.031**⁎	**0.049**⁎	1.61	(0.82, 3.14)	0.167	0.167	12	1.29	(0.70, 2.36)	0.412	0.549	—			
5	Adult sexual abuse (ever)	2.33	(1.35, 4.04)	**0.002**⁎⁎	**0.005**⁎⁎	1.98	(1.11, 3.54)	**0.021**⁎	0.053	13	1.87	(1.09, 3.19)	**0.022**⁎	0.088	1.61	(0.92, 2.82)	0.095	0.143
6	Adult physical abuse (ever)	2.32	(1.31, 4.12)	**0.004**⁎⁎	**0.005**⁎⁎	1.85	(1.01, 3.40)	**0.048**⁎	0.080	14	1.74	(1.00, 3.03)	**0.050**⁎	0.133	1.45	(0.81, 2.60)	0.214	0.214
7	Adult sexual abuse (past year)	1.39	(0.72, 2.70)	0.329	0.329	—				15	1.17	(0.61, 2.26)	0.631	0.631	—			
8	Adult physical abuse (past year)	1.42	(0.72, 2.79)	0.310	0.329	—				16	1.46	(0.75, 2.85)	0.261	0.418	—			

*Source:* The ESSENCE Project 2015–2018.

*Notes:* Boldfaces indicate statistical significance.

Adjusted models include the bivariate association and the confounders of age, high school education, and total number of lifetime sexual partners. A 0.05 false discovery rate level was applied to Models 1–16.

BV, bacterial vaginosis.

⁎ *p<*0.05, **p<0.01, and ***p<0.001).

**Table 3:** Logistic Regression Models of Cumulative Violence and BV Infection as Predictors of STIs Among Black Women in Baltimore, Maryland (N=230)

Model		Lifetime STI (yes/no)								Model	Past-year STI (yes/no)							
		Unadjusted model				Adjusted model					Unadjusted model				Adjusted model			
		OR	95% CI	*p*-value	Adjusted *p*-value	AOR	95% CI	*p*-value	Adjusted *p*-value		OR	95% CI	*p*-value	Adjusted *p*-value	AOR	95% CI	*p*-value	Adjusted *p*-value
1	Cumulative violence (continuous)	1.05	(0.91, 1.21)	0.514	0.514	—				6	0.95	(0.84, 1.07)	0.415	0.533				
2	Cumulative violence (categorical)									7								
	0	ref									ref							
	1–6	1.49	(0.80, 2.75)	0.208	0.277	—					0.87	(0.51, 1.48)	0.600	0.600	—			
3	BV infection (ever)	2.90	(1.57, 5.34)	**0.001**⁎⁎⁎	**0.004**⁎⁎	2.76	(1.45, 5.22)	**0.002**⁎⁎	**0.004**⁎⁎	8	1.80	(1.07, 3.05)	**0.028**⁎	0.056	2.10	(1.19, 3.70)	**0.011**⁎	**0.017**⁎
4	BV infection (past year)	2.25	(1.20, 4.21)	**0.011**⁎	**0.022**⁎	2.16	(1.14, 4.10)	**0.018**⁎	**0.018**⁎	9	2.54	(1.49, 4.34)	**0.001**⁎⁎⁎	**0.004**⁎⁎	3.00	(1.70, 5.31)	**0.000**⁎⁎⁎	**0.003**⁎⁎
5	Interaction model																	
	Cumulative violence (continuous)	0.87	(0.72, 1.05)	0.148		0.86	(0.71, 1.05)	0.137			—				—			
	BV infection (ever)	1.69	(0.81, 3.54)	0.164		1.63	(0.77, 3.48)	0.205			—				—			
	Cumulative violence (continuous)^×^ BV infection (ever)	1.44	(1.05, 1.98)	**0.022**⁎		1.45	(1.05, 1.99)	**0.024**⁎			—				—			

*Source:* The ESSENCE Project 2015–2017.

*Notes:* Boldfaces indicate statistical significance.

Adjusted models include the bivariate association and the confounders of age, high school education, and total number of lifetime sexual partners. A 0.05 false discovery rate level was applied to Models 1–4 and 6–9.

⁎ *p<*0.05, **p<0.01, and ***p<0.001). BV, bacterial vaginosis; STI, sexually transmitted infection.

Finally, we estimated models to test whether BV infection moderated the association between cumulative lifetime violence and STIs (Table 3: Model 5). For the moderation model, we estimated an interaction between cumulative violence and BV diagnosis to test our hypothesis that the association between cumulative violence and STI diagnosis is stronger for women with a BV diagnosis than for women without a BV diagnosis.

## RESULTS

Table 1 reports the sociodemographic, cumulative violence, and STI characteristics of the total and stratified samples (stratified by lifetime versus past-year BV diagnosis). The median age was 25 (IQR=21–30) years. Most women had at least a high school education (84%), were formally employed (61%), had an individual annual income <$10,000 (58%), identified as heterosexual (85%), and were single (69%). Women had a median of 2 sexual partners in their lifetime (IQR=1–2). Women had a median cumulative violence score of 0 (IQR=0–4), with 40% reporting at least 1 experience of violence, 24% reporting child sexual abuse, 24% reporting child physical abuse, 40% reporting adult sexual abuse, 33% reporting adult physical abuse, 20% reporting past-year adult sexual abuse, and 19% reporting past-year adult physical abuse. A large proportion (73%) of women reported lifetime STI diagnosis, whereas 46% reported past-year STI diagnosis.

Women with lifetime BV diagnosis were significantly older; reported more experiences of child physical abuse, adult sexual abuse, and adult physical abuse; had a higher cumulative violence score; and had more lifetime and past-year STIs than women with no prior BV infection. The same held for women with past-year BV diagnosis, except that frequency of child physical abuse and cumulative violence score did not significantly differ from those reported by women with no past-year BV diagnosis.

Table 2 reports findings from logistic regression models assessing the impacts of cumulative violence on BV diagnosis. Compared with women reporting no cumulative violence exposure, women with 1–6 cumulative violence exposures had increased odds of ever having a BV infection (AOR=1.98; 95% CI=1.10, 3.54), but these were marginally significant once adjusting for multiple comparisons using 0.5 FDR. Among the cumulative violence types, adult sexual abuse (AOR=1.98; 95% CI=1.11, 3.54) was significantly associated with increased adjusted odds of having a lifetime BV diagnosis, but this was marginally significant once adjusting for multiple comparisons using 0.5 FDR.

Table 3 reports findings from logistic regression models of the association of cumulative violence and BV infection on STIs. Lifetime BV diagnosis (AOR=2.76; 95% CI=1.45, 5.22) and past-year BV diagnosis (AOR=2.16; 95% CI=1.14, 4.10) were both associated with significantly increased odds of lifetime STI diagnosis. In addition, lifetime BV diagnosis (AOR=2.10; 95% CI=1.19, 3.70) and past-year BV diagnosis (AOR=3.00; 95% CI=1.70, 5.31) were significantly associated with past-year STI diagnosis.

**Figure 1:**
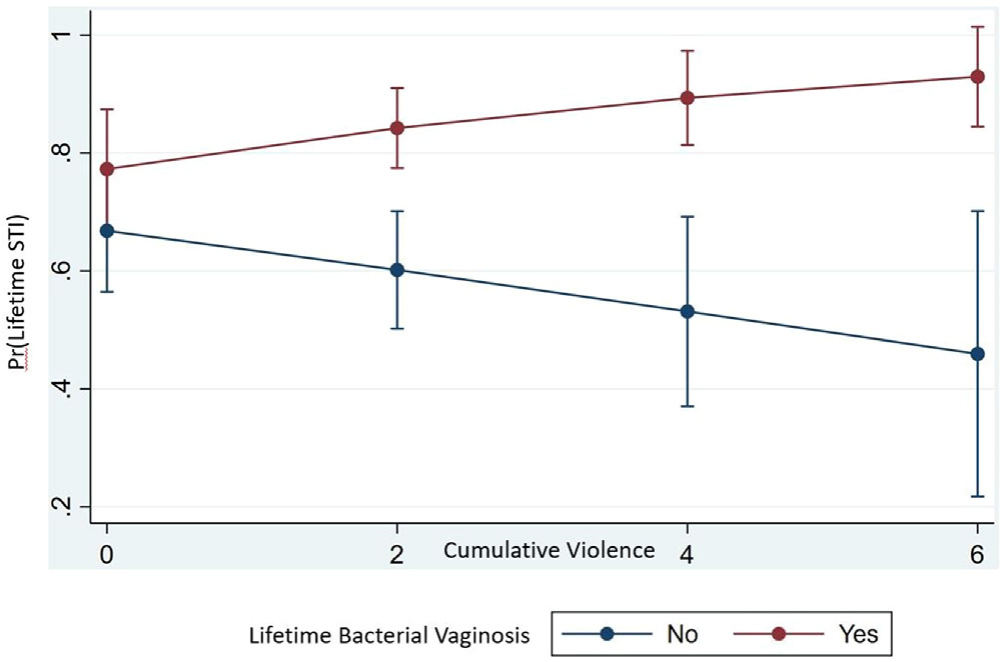
Predictive margins with 95% CIs of the interaction of cumulative violence and lifetime bacterial vaginosis. STI, sexually transmitted infection.

We then examined whether lifetime BV moderates the association between cumulative violence and STIs (Table 3). Figure 1 shows the positive relationship between cumulative violence and lifetime STIs for women ever reporting BV diagnosis. Similarly, it demonstrates a negative relationship between cumulative violence and lifetime STI for women who did not ever experience BV. The directionality of the association between cumulative violence and lifetime STI differed significantly between women reporting lifetime BV and those not reporting BV.

## DISCUSSION

This study examined the associations between cumulative violence, BV, and STIs among Black women. From our analysis, we had 4 key findings: (1) women with cumulative violence exposure had greater odds of lifetime BV diagnosis, (2) sexual abuse during adulthood was associated with greater odds of lifetime BV diagnosis, (3) BV diagnosis was significantly associated with increased odds of STI diagnosis, and (4) the impact of cumulative violence on lifetime STIs is moderated by the presence of lifetime BV diagnosis.

Cumulative violence significantly increased the odds of reporting a lifetime BV diagnosis. According to MST, Black women may face more cumulative violence owing to their intersecting minority statuses (e.g., race, gender). Cumulative violence over their life course can be a significant, chronic stressor that may cause physiologic changes and altered inflammatory response, thereby increasing the risk for BV.^25^^,^^34^, ^35^, ^36^, ^37^ Existing literature also identified stressful life events, including violence, as significant predictors of BV. One study found that each additional stressful life event reported resulted in an estimated 13% increase in BV risk for Black women (95% CI=1.04, 1.19).^38^ In addition, cumulative violence may also significantly contribute to increased risk behaviors, such as condomless sex and multiple partners, associated with increased BV risk.^5^

Adult sexual abuse and reporting any cumulative violence were associated with greater odds of lifetime BV, controlling for other factors. Identifying specific violence types associated with BV is essential for developing targeted interventions to overcome these increased risks. This abuse may lead to toxic stress and physiologic changes that create an environment that increases the risk for BV.^30^^,^^39^, ^40^, ^41^ In addition, sexual abuse may increase the risk of BV owing to vaginal trauma.^41^^,^^42^ Our findings support the criticality of addressing violence and screening for BV among abused women, especially Black women who face additional minority stressors.

Our study found that BV was significantly associated with both increased odds of lifetime and past-year STI infection, an association also found in the Galvin and Cohen review.^5^ Our findings are similar to existing research that examined a cohort of women in India and found that current BV infection was significantly associated with incident STI infection.^43^ STI risk reduction interventions should also consider how to lower rates of BV.^44^^,^^45^

We found that BV infection moderates the association between cumulative violence and lifetime STI diagnosis. Specifically, we observed a positive association between cumulative violence and lifetime STI among women with a history of BV infection, whereas a negative association was found among women without a BV history. The interaction between cumulative violence and BV may partially explain the disproportionate STI and HIV rates in Black women. Although we cannot determine temporality between BV and STIs, our cross-sectional results provide support to prior studies in which BV led to inflammatory and physiologic changes that increased susceptibility to STIs.^21^ This identifies a potentially important mechanism by which we can attenuate cumulative violence's adverse sexual health effects. Our findings support recommendations to address BV in efforts to reduce rates of STIs for Black women.^46^

### Limitations

This study has strengths but is also not without limitations. Strengths of this study include the comprehensiveness of our cumulative violence variable in which we identified different types of cumulative violence events. We gain better insight into how different types of violence and their timing over the life course are associated with BV and STIs, especially in cities such as Baltimore with a high concentration of STIs. In terms of limitations, there may have been reporting bias in self-reported measures, particularly with BV. The number of women with previous BV infection may be higher than reported because BV can go undiagnosed, and there can be issues with recall accuracy. Another limitation is that our measure of IPV centers on violence perpetrated by male sex partners. This does not fully encompass the experiences of nonheterosexual women. We also do not have a direct measure to evaluate stress, so we are unable to evaluate explicitly the role that chronic stress plays in the relationship between IPV and BV. In addition, this study's cross-sectional design does not allow a temporal examination of relationships, limiting our conclusions.

## CONCLUSIONS

The MST highlights the toxic stress that Black women in the U.S. face at the confluence of race, gender, and violence. That stress conveys a risk for adverse mental and physical effects.^25^^,^^27^ Cumulative violence across the life course can add to these stressors and have deleterious physiologic effects on immune function,^5^^,^^26^ and can potentially increase the risk for BV infection. This study supports efforts to limit the disproportionately high rates of BV and STIs among Black women, especially survivors of violence. Our results support the need to further study whether leveraging positive coping responses, such as resilience and social support, reduces the negative impacts of BV and STIs among Black women who experience violence-induced stress across their lifetime.

## CRediT authorship contribution statement

**Yordanos Tesfai:** Conceptualization, Writing – original draft. **Marguerite B. Lucea:** Writing – review & editing. **Erica Chan:** Writing – review & editing. **Theresa Asuquo:** Writing – original draft, Writing – review & editing. **Helen Zhu:** Writing – review & editing. **Tommi L. Gaines:** Formal analysis, Supervision. **Jacquelyn C. Campbell:** Conceptualization, Data curation, Funding acquisition, Writing – review & editing. **Jamila K. Stockman:** Conceptualization, Data curation, Funding acquisition, Writing – review & editing. **Kiyomi Tsuyuki:** Conceptualization, Formal analysis, Writing – original draft.
